# Global, regional, and national burden of disease study of atrial fibrillation/flutter, 1990–2019: results from a global burden of disease study, 2019

**DOI:** 10.1186/s12889-022-14403-2

**Published:** 2022-11-03

**Authors:** Hong Li, Xuejing Song, Yi Liang, Xue Bai, Wu-Sha Liu-Huo, Chao Tang, Wen Chen, Lizhi Zhao

**Affiliations:** 1 Southwest Medical University, Lu Zhou, China; 2 The Affiliated Traditional Chinese Medicine Hospital of Southwest Medical University, Lu Zhou, China

**Keywords:** Atrial fibrillation/flutter, Incidence, Prevalence, Deaths, DALYs, Age-standardised rate, Risk factor

## Abstract

**Background:**

Data from the Global Burden of Disease, Injury, and Risk Factor Study 2019 (GBD 2019) was used to assess the burden and change in prevalence, incidence, deaths, disability-adjusted life years, and risk factors for atrial fibrillation/flutter in 204 countries and territories between 1990 and 2019.

**Methods:**

Incidence, prevalence, deaths, disability-adjusted life years (DALYs), and their age-standardized rates of AF/AFL were analyzed by age, sex, socio-demographic index (SDI), and human development index (HDI) using the Global Burden of Disease study 2019 (GBD2019) results，and risk factors for AF/AFL (mainly high systolic blood pressure, high body-mass index, alcohol use, smoking and diet high in sodium) were differentially analyzed.

**Results:**

There are 59.70 million (95% uncertainty interval (UI) 45.73–75.29 million) AF/AFL patients worldwide in 2019, with 4.72 million (95% uncertainty interval (UI) 3.64–5.96 million) new cases and 0.315 million deaths (95% uncertainty interval (UI) 0.268–0.361 million) and 8.39 million disability-adjusted years (95% uncertainty interval (UI) 6.69–10.54 million). The highest risk factor for deaths, DALYs attributable to AF/AFL in 2019 was high systolic blood pressure, high body-mass index, alcohol use, smoking, and diet high in sodium. It is estimated that between 2030 and 2034, the total incidence of male AF/ AFL will be 16.08 million, and the total number of deaths will be 1.01 million. For females, the total number of incidence is 16.85 million, and the total number of deaths is 1.49 million.

**Conclusions:**

AF/AFL remains a major global public health problem, although the ASR of prevalence, incidence, and DALY at the worldwide level showed a decreasing trend from 1990 to 2019(the ASR of deaths increased slightly). However, the unfavorable trend observed in this study in countries with lower SDI suggests that current prevention and treatment strategies should be reoriented. Some countries should develop more targeted and specific strategies to prevent the increase of AF/AFL.

**Supplementary Information:**

The online version contains supplementary material available at 10.1186/s12889-022-14403-2.

## Introduction

Atrial fibrillation is the most common sustained arrhythmia in the world and is one of the leading causes of stroke, heart failure, sudden deaths, and cardiovascular disease, these adverse events can result in high health care costs and pose a significant public health burden [1, 2]. The occurrence of atrial fibrillation/atrial flutter is complex, and the main risk factors include hypertension, smoking, alcohol consumption, high sodium intake, obesity, etc. Socio-demographic factors such as population, age, race, and socio-economic factors such as income, education, and health care resources are also important influences on the burden of AF/AFL. At the same time, hypertension is the most crucial factor leading to atrial fibrillation [3–5]. The number of people with atrial fibrillation worldwide increased rapidly between 1990 and 2017, rising from 19.1 million to 37.6 million, and is expected to increase further in the future [6]. The public health burden on global health systems is enormous, and the consumption of social resources and costs are increasing significantly. At the same time, the development of AF/AFL is closely related to the socio-economic level and cultural level with the globalization of the economy, some countries or regions have the contradiction of dramatic increase in economic income and low cultural and educational levels, resulting in increased incidence and deaths, and a higher disease burden [7, 8]. In countries with higher levels of economic development, more adequate health care resources, higher literacy and education levels, the incidence and prevalence of AF/AFL, although higher than in developing countries, have better diagnostic and treatment measures and longer survival years [9, 10]. No systematic studies have been conducted since the Global Burden of Disease, Injury, and Risk Factor Study 2019(GBD2019) for a comprehensive update on epidemiological trends in AF/AFL. Based on the Global Burden of Disease, Injury, and Risk Factor Study 2019 [11, 12] data, this study intends to conduct a systematic and comprehensive analysis of the disease burden of AF/AFL and assess the trend of AF/AFL occurrence in terms of global, regional, and national AF/AFL prevalence, incidence, deaths, disability-adjusted life years and risk factors (mainly focused on high systolic blood pressure, high body-mass index, alcohol use, smoking and diet high in sodium). Our findings may help national health authorities gain a comprehensive understanding of the global burden of AF/AFL and develop rational prevention and treatment policies, allocate health resources, and improve the prognosis of AF/AFL patients.

## Materials and methods

### Data sources

The GDB2019 study is an update and expansion of GDB2017, which analyzed and calculated epidemiological data for 204 countries, 22 regions, 369 diseases, and 87 risk factors, including incidence, prevalence, deaths, and disability-adjusted life years, through the collection and collation of published literature, surveys, and epidemiological information by researchers in multiple countries [11]. 204 countries were classified into five regions based on the Socio-Demographic Index (SDI), including low SDI, low-middle SDI, middle SDI, high-middle SDI, and high SDI [11]. The SDI is a composite indicator used to assess the development status of each location year. It consists of the geometric mean of three common indicators (lagging per capita income distribution, average educational attainment of the population aged 15 years or older, and total fertility rate for those under 25 years of age. Regions or countries with an SDI of 0 have the theoretical lowest level of health-related development, while regions or countries with an SDI of 1 have the theoretical maximum level of development. In addition, the globe was geographically divided into 21 regions, and in this study, AF/AFL incidence, prevalence, deaths, DALYs, and their corresponding age-standardized rates (ASR) were collected by the Global Health Data Exchange (GHDx) query tool (http://ghdx.healthdata.org/gbd-results-tool). Annual deaths and DALYs attributable to 87 risk factors were also available from the GBD results Tool. ASRs were calculated using the GBD World Population Criteria. The uncertainty interval (UI) for all estimates was 95%.

### Atrial fibrillation/flutter definition

The definitions of AF/AFL are all based on the International Classification of Diseases (ICD) diagnostic criteria. The relevant ICD9 and ICD11 incidence and death codes for AF/AFL are atrial fibrillation (BC81.3Z, BC81.32, BC81.33, BC81.31, BC81.3Y BC81.30), atrial flutter (BC81.2Z, BC81.20).

### Attributable risk factors

Attributable risk factors for AF/AFL include high systolic blood pressure, high body-mass index, smoking, alcohol use, and diet high in sodium. Percentages and DALYs for AF/AFL-related deaths are available in the GBD Outcomes Tool (https://vizhub.healthdata.org/gbd-results/). Details of the definitions of these risk factors and methods for quantifying the percentage contribution of these risk factors to AF deaths have been published elsewhere [5, 13, 14].

### Statistical analyses

This study used age-standardized rates (ASR) for incidence, prevalence, deaths, DALYs, and estimated annual percentage change (EAPC) in prevalence to quantify the global burden of AF/AFL [15]. Standardization is necessary when comparing several populations with different age structures or changes in age structure of the same population over time. The units of the standardized rate are per 100,000 people, and ASR trends can be a good proxy for shifts in disease patterns within a population and can serve as a cue for changing risk factors. Thus, we can use ASR to develop more targeted prevention and treatment strategies. EAPC is a summary and widely used measure of ASR trends over specific time intervals. The regression line conforms to the natural logarithm of the ratio, y = α + βx + ε, where y = ln (ASR) and x = calendar year. ε is the error, and EAPC is reported with a 95% confidence interval (CI). The ASR is considered to be on an upward trend when both the EAPC estimates and their lower 95% CI are > 0. Conversely, when both the EAPC estimate and its upper 95% CI were < 0, the ASR was considered to be in a downward trend. Otherwise, ASR was considered stable over time, and Pearson’s correlation coefficient was used to evaluate the relationship between ASR and different SDI regions. In addition, to explore the influences of EAPC, we evaluated the association between EAPC and ASR (1990) and the Human development index 2019 (HDI 2019) separately at the national level. Finally, hierarchical cluster analysis classified countries and regions into four categories (a: significant increase; b: slight increase; c: remained stable or slightly decreased; d: significant decrease). This study applies decomposition analysis to describe factors associated with changes in DALYs between 1990 and 2019. the overall variation in DALYs was constructed through three factors: (1) age structure, (2) population growth, and (3) age-specific DALYs rates (epidemiological changes). To better predict the future global disease burden of AF/AFL, this study used Nordpred age-period-cohort (APC) analysis to predict the total incidence and death of AF/AFL every five years [16]. All data statistical analysis and visualization were performed using the R program (version 4.1.2, RStudio) and GraphPad Prism software (version 9.3.1). *P*-values less than 0.05 were considered to be statistically significant.

## Results

### 1990–2019 global AF/AFL disease burden trends

**Fig. 1:**
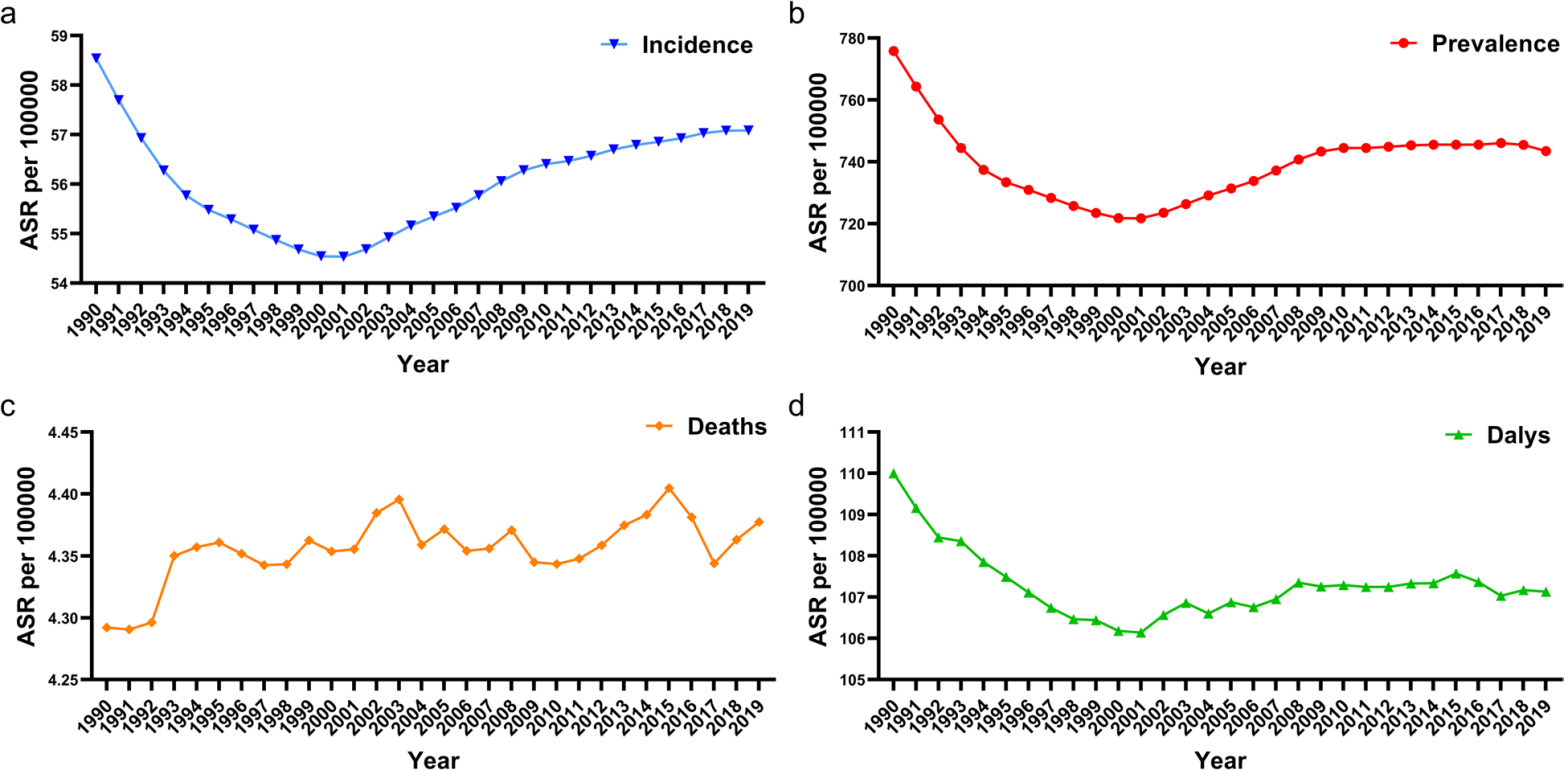
Age-standardized rate of incidence, prevalence, deaths, and DALYs change curves for AF/AFL patients from 1990 to 2019(a: Incidence;b: Prevalence;c: Deaths;d: DALYs)

The global disease burden of AF/AFL increased significantly from 1990 to 2019 (Table 1). The number of incidences increased from 2.134 million (1.764–2.951 million) in 1990 to 4.720 million (3.644–5.962 million) in 2019, with a 103.9% increase. The standardized prevalence rate decreased from 58.5 per 100,000 (44.9–74.2) to 57.1 per 100,000 (44.1–71.9). The number of prevalence increased from 2.8286 million (2.1493–3.6162 million) in 1990 to 59.695 million (45.730–75.287 million) in 2019, with a 111.0% increase. The standardized prevalence rate decreased from 775.9/100,000 (592.4–990.8) to 743.5/100,000 (571.2–938.3). The number of global AF/AFL deaths increased from 0.117 (0.104–0.138 million) in 1990 to 0.315 (0.268–0.361 million) in 2019, which increased by 169.2%. The standardized deaths rate increased from 4.3 per 100,000 (3.7–5.1) to 4.4 per 100,000 (3.7–5.0) DALYs increased from 3.788 million (2.961–4.832 million) in 1990 to 8.393 million (6.694–10.541 million) in 2019, an increase of 121.6%. The rate of age-standardized DALYs decreased from 110.0 per 100,000 (87.7–139.2) to 107.1/100,000 (86.1–133.7). From 1990 to 2019, the study found an interesting phenomenon: the ASR of incidence, prevalence, and DALYs all showed a gradual decrease until 2001 and reached a minimum in 2001, and the ASR of incidence showed a gradual increase after 2001. Meanwhile, the ASR of prevalence gradually increased after 2001 and leveled off after 2010, while standardized DALYs did not increase significantly afterward (Fig. 1). Fig. S1 and Table S1 -S2 show the Nordpred APC model estimated that between 2030 and 2034, the AF/ AFL total incidence of males will be 16.08 million, and the total number of deaths will be 1.01 million. For females, the total number of incidence will be 16.85 million, and the total number of deaths will be 1.49 million.

**Table 1:** The prevalence cases and age-standardized prevalence of AF/AFL in 1990 and 2019, and its temporal trends from 1990 to 2019

Characteristics	1990		2019		1990–2019
	Prevalence cases No.× 10^5^ (95% UI)	ASR per 100,000 No. (95% UI)	Prevalence cases No.× 10^5^ (95% UI)	ASR per 100,000 No. (95% UI)	EAPC No. (95% CI)
Global	282.9 (214.9–361.6)	775.9 (592.4–990.8)	597.0 (457.3–752.9)	743.5 (571.2–938.3)	0.01 (− 0.06–0.08)
High SDI	100.6 (76.6–127.4)	947.6 (727.7–1199.4)	175.7 (138.7–217.2)	895.7 (707.8–1104.9)	0.16 (0.01–0.31)
High-middle SDI	83.1 (63.0–107.0)	818.6 (625.2–1042.3)	159.7 (121.3–203.7)	780.2 (594.1–996.5)	− 0.14(− 0.16--0.12)
Middle SDI	56.3 (42.6–72.2)	642.3 (486.6–819.1)	155.1 (117.2–199.6)	672 (507.7–859.3)	0.19 (0.13–0.25)
Low-middle SDI	33.1 (25.0–42.8)	659.3 (500.5–843.5)	83.8 (63.2–107.3)	677 (511.8–863.1)	0.13 (0.11–0.15)
Low SDI	9.6 (7.2–12.4)	492.3 (374.4–630.2)	22.5 (17.1–29.1)	514.2 (390.1–658.4)	0.16 (0.15–0.17)
Andean Latin America	0.25 (0.19–0.32)	140.5 (106.1–181)	0.85 (0.64–1.1)	158.1 (119.6–202.1)	0.56 (0.47–0.64)
Australasia	3.1 (2.3–3.9)	1300.6 (982.4–1657.3)	6.2 (4.7–7.9)	1212.2 (927.8–1530.3)	−0.18 (− 0.23--0.13)
Caribbean	0.83 (0.63–1.1)	336.1 (254.2–433.9)	1.8 (1.4–2.3)	344.3 (262.5–440.9)	0.11 (0.1–0.13)
Central Asia	3.8 (2.8–4.8)	857 (649.7–1097.6)	5.9 (4.4–7.6)	893.6 (680.6–1145.9)	0.19 (0.17–0.21)
Central Europe	15.0 (11.3–19.3)	1026.8 (778.7–1318)	22.1 (16.6–28.4)	1001.8 (756.8–1282.3)	0.03 (−0.05–0.12)
Central Latin America	2.7 (2.0–3.5)	366.2 (278.5–472)	8.3 (6.3–10.7)	368.6 (280.4–474.2)	0.04 (0.03–0.05)
Central Sub-Saharan Africa	0.71 (0.53–0.92)	394.6 (297.9–507.7)	1.7 (1.3–2.1)	384.5 (291.3–497.7)	−0.11 (− 0.13--0.09)
East Asia	51.1 (38.5–66.0)	687.8 (520.9–881.5)	143.4 (108.3–186.0)	723.4 (546.9–923.9)	0.22 (0.1–0.35)
Eastern Europe	26.0 (19.6–33.5)	946.3 (722.9–1209.9)	37.0 (28.1–47.6)	1046.9 (797.4–1344.7)	0.42 (0.39–0.45)
Eastern Sub-Saharan Africa	1.4 (1.0–1.8)	226.9 (172.3–293)	3.2 (2.4–4.1)	233.2 (177.4–300.8)	0.2 (0.14–0.26)
High-income Asia Pacific	7.5 (5.7–9.5)	376.6 (290.8–477.2)	13.5 (10.5–17.1)	312.1 (242–394.5)	−1.31 (−1.62--1)
High-income North America	42.9 (32.7–54.7)	1173.6 (898.8–1486.1)	86.2 (70.2–104.4)	1322.2 (1074.9–1600.7)	1.21 (0.86–1.55)
North Africa and Middle East	7.2 (5.4–9.2)	498.2 (379.2–638.2)	19.0 (14.5–24.2)	506.4 (385.7–648.5)	−0.04 (−0.08--0.01)
Oceania	0.17 (0.13–0.21)	707.9 (534.1–905.9)	0.41 (0.31–0.53)	733.5 (552.3–942.1)	0.1 (0.09–0.12)
South Asia	34.2 (25.8–44.2)	746.3 (566.1–953)	96.6 (72.5–124.0)	763.9 (577.7–972.9)	0.07 (0.07–0.08)
Southeast Asia	16.4 (12.5–21.2)	755.7 (570.4–967.1)	42.6 (32.2–54.5)	781.8 (589.3–998.6)	0.13 (0.12–0.14)
Southern Latin America	2.3 (1.7–2.9)	504.7 (378.9–645.2)	4.5 (3.5–5.7)	529.6 (400.6–675.9)	0.14 (0.08–0.21)
Southern Sub-Saharan Africa	1.1 (0.85–1.4)	455.5 (347.2–586)	2.3 (1.7–2.9)	449.4 (340.6–578.6)	−0.03 (−0.04--0.03)
Tropical Latin America	4.1 (3.1–5.3)	518 (391.9–666.7)	12.5 (9.5–16.1)	536 (408.5–690.8)	0.66 (0.47–0.85)
Western Europe	59.1 (45.0–75.1)	996 (764.6–1265.3)	82.1 (62.5–103.8)	886.7 (680.2–1124.2)	−0.23 (− 0.27--0.19)
Western Sub-Saharan Africa	3.3 (2.5–4.2.3)	430.3 (326.8–550.3)	7.0 (5.3–9.0)	441.6 (334.5–568.1)	0.09 (0.06–0.13)

*ASR* Age-standardized rate, *CI* Confidence interval, *EAPC* Estimated annual percentage change, *UI* Uncertainty interval

### Global age distribution of AF/AFL disease burden in 2019

**Fig. 2:**
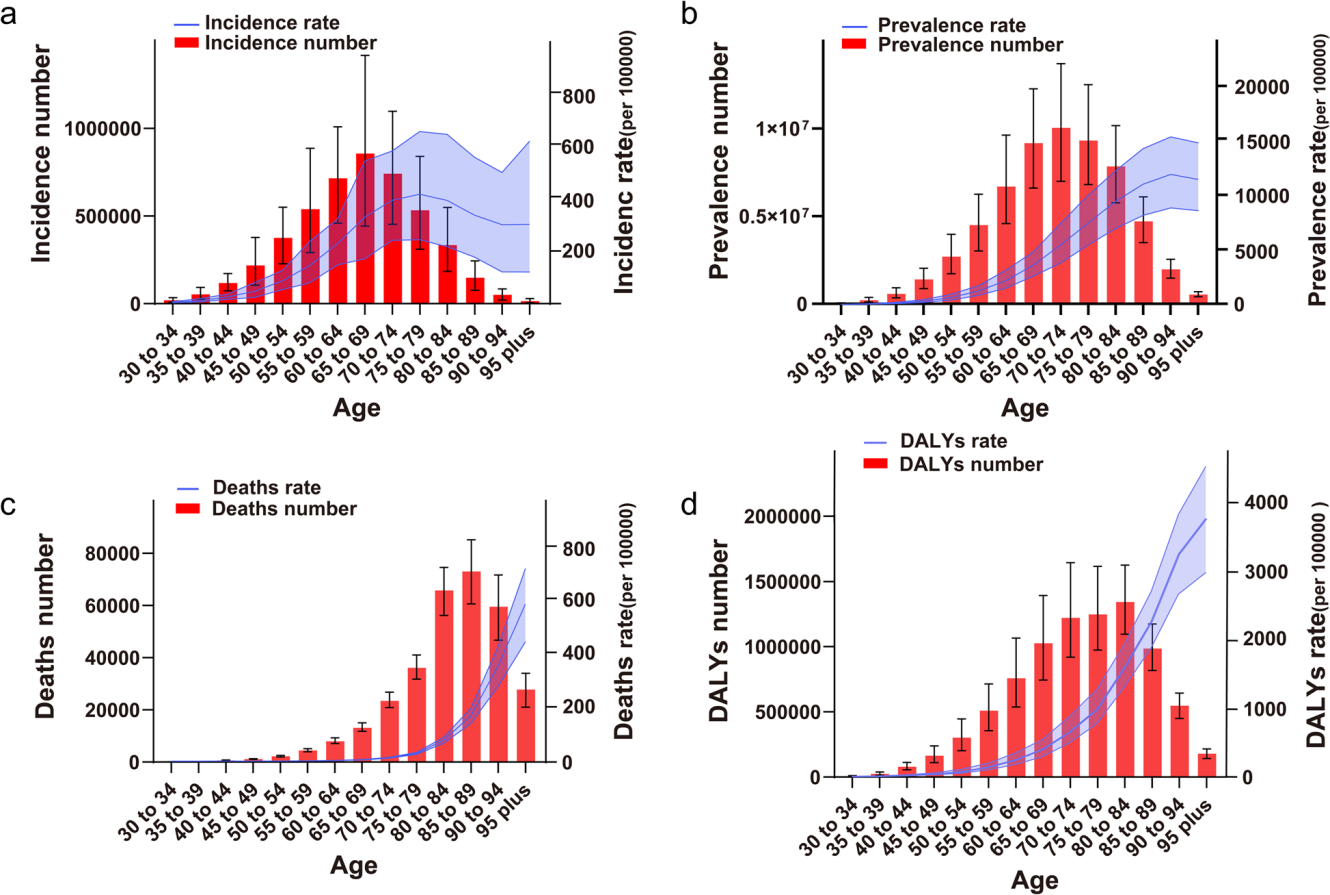
The number of AF/AFL patients with incidence, prevalence, deaths, and DALYs and their ASR in each age group (per 5-year), the number on the left side and the ASR on the right side

The global prevalence, incidence, deaths, and DALYs ratio of AF/AFL showed an overall increasing trend with age (Fig. 2, Table S3) and the incidence of AF/AFL increased significantly with age, peaking between 75 and 79 years and gradually decreasing after 80 years (Fig. 2a). The prevalence rate gradually increased with age, peaking between 90 and 94 years and then dropping (Fig. 2b). The death rate showed an increasing trend with age trend (Fig. 2c), and the rate of DALYs tended to increase with age (Fig. 2d).

### Gender distribution of the global AF/AFL disease burden

**Fig. 3:**
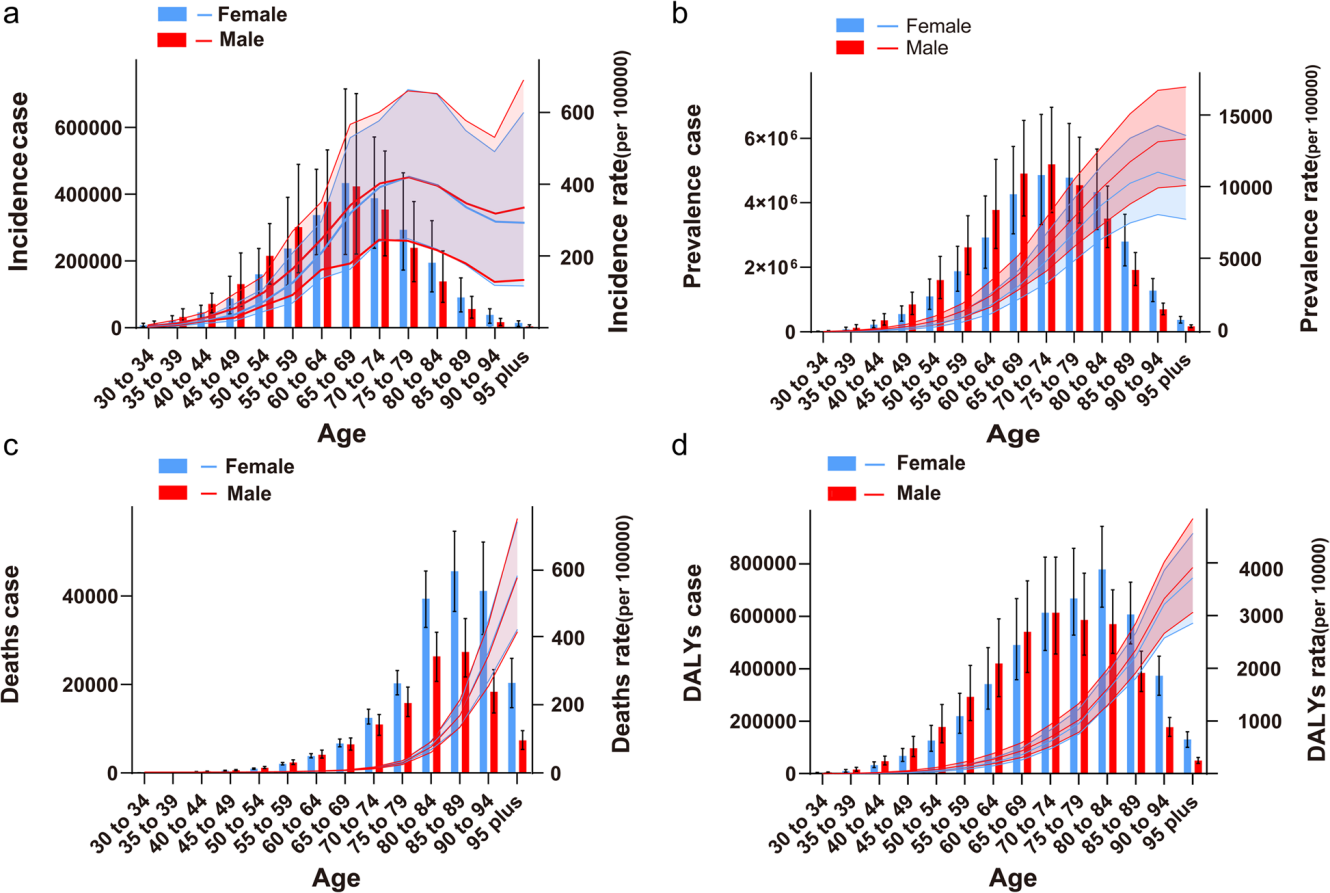
The number of AF/AFL patients of each age group (per 5-year cohort) in males and females with incidence, prevalence, death, and DALYs and their ASR, with the number on the left side and the ASR on the right side, with females in blue and males in red

From 1990 to 2019, the number of incidence, prevalence, deaths, DALYs, and their ratios for both males and females showed an increasing trend over time. In terms of overall numbers and trends, the overall burden of disease was slightly higher for males than females (Fig. S2). In 2019, there are significant differences in disease burden between males and females in different age groups, with the female having a greater overall number of incidence than males after 65 years of age, a greater number of prevalence than males after 75 years of age, a significantly higher number of deaths than males after 65 years of age, and a higher total number of DALYs than males after 70 years of age (Fig. 3). From Tables S4-S5, the incidence rates of AF/AFL in 2019 was 60.8/100,000 (46.6–77.1) for females and 61.2/100,000 (47.3–77.6) for males; the prevalence rates was 750.1/100,000 (575.4–947.0) for females and 780.3/100,000 (603.6–987.7) for males. Deaths rates was 5.0/100,000 (4.2–5.8) for female and 3.1 /100,000 (2.5–3.8) for male; DALYs rates was 115.1/100,000 (143.6–92.7) for female and 101.9 /100,000 (79.6–130.1) for male.

### Global distribution of AF/AFL disease burden across regions and countries

**Fig. 4:**
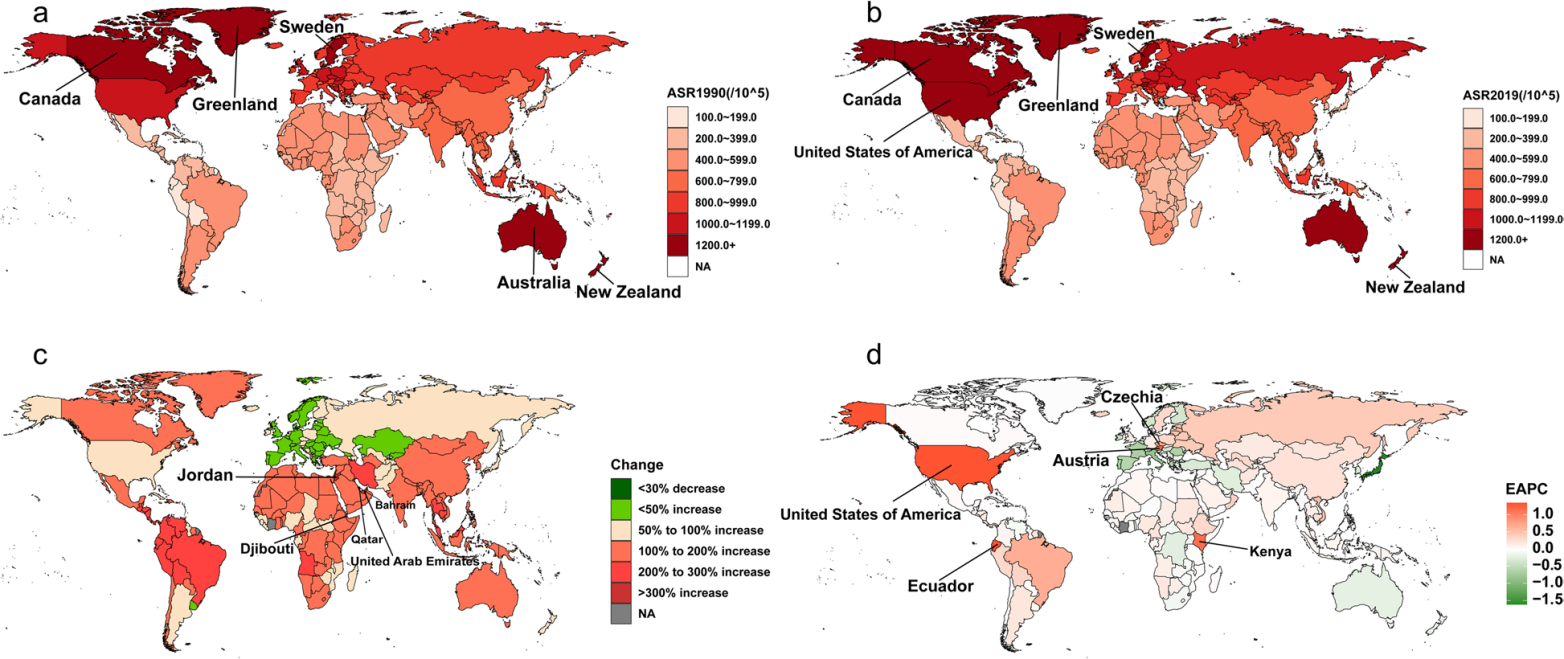
Global age-standardized prevalence of AF/AFL for both sexes in 204 countries and territories. **a** Age-standardized prevalence of AF/ AFL in 1990, **b** age-standardized prevalence of AF/AFL in 2019,**c** the relative change in prevalence of AF/AFL between 1990 and 2019, and **d** The EAPC of the prevalence of AF/AFL between 1990 and 2019. AF/AFL, atrial fibrillation/atrial flutter; EAPC, estimated annual percentage change

The geographic distribution heat map (Fig. 4) shows that the disease burden of AF/AFL in 1990 varies significantly by country. The top five countries in terms of prevalence were China (4,927,000), the United States of America (3,881,000), India (2,691,000), the Russian Federation (1,635,000) and Germany (1,396,000). After correcting for age and demographic factors, the top five prevalence countries were New Zealand (1329.1 per 100,000), Sweden (13.09 per 100,000), Australia (1294.9 per 100,000), Canada (1235.9 per 100,000), and Greenland (1227.8 per 100,000) (Fig. 4a). The disease burden of AF/AFL in 2019 varied significantly between countries or regions. The top five countries in terms of prevalence were China (13.883 million), India (7.996 million), the United States of America (7.729 million), the Russian Federation (2.774 million), and Germany (1.824 million). After adjusting for age and population, the top five countries in terms of prevalence were the United States of America (1331.4/100,000), Sweden (1279.0/100,000), Canada (1248.5/100,000), Greenland (1231.3/100,00), and New Zealand (1223.2/100,00) (Fig. 4b). The top five countries in the world in terms of prevalence growth rate by country and region from 1990 to 2019 were the United Arab Emirates (796.1%), Qatar (790.0%), Bahrain (471.5%), Jordan (415.9%), and Djibouti (381.5%) (Fig. 4c). The top 5 countries in terms of change in prevalence (EAPC) after correcting for age and population factors were the United States of America (1.3), Ecuador (1.3), Austria (1.2), Kenya (1.1), and Czechia (0.7) (Fig. 4d). Overall, the disease burden of AF/AFL was higher in countries with larger populations.

### Relationship between AF/AFL disease burden and level of socio-economic development

#### Trends in AF/AFL disease burden by SDI level

**Fig. 5:**
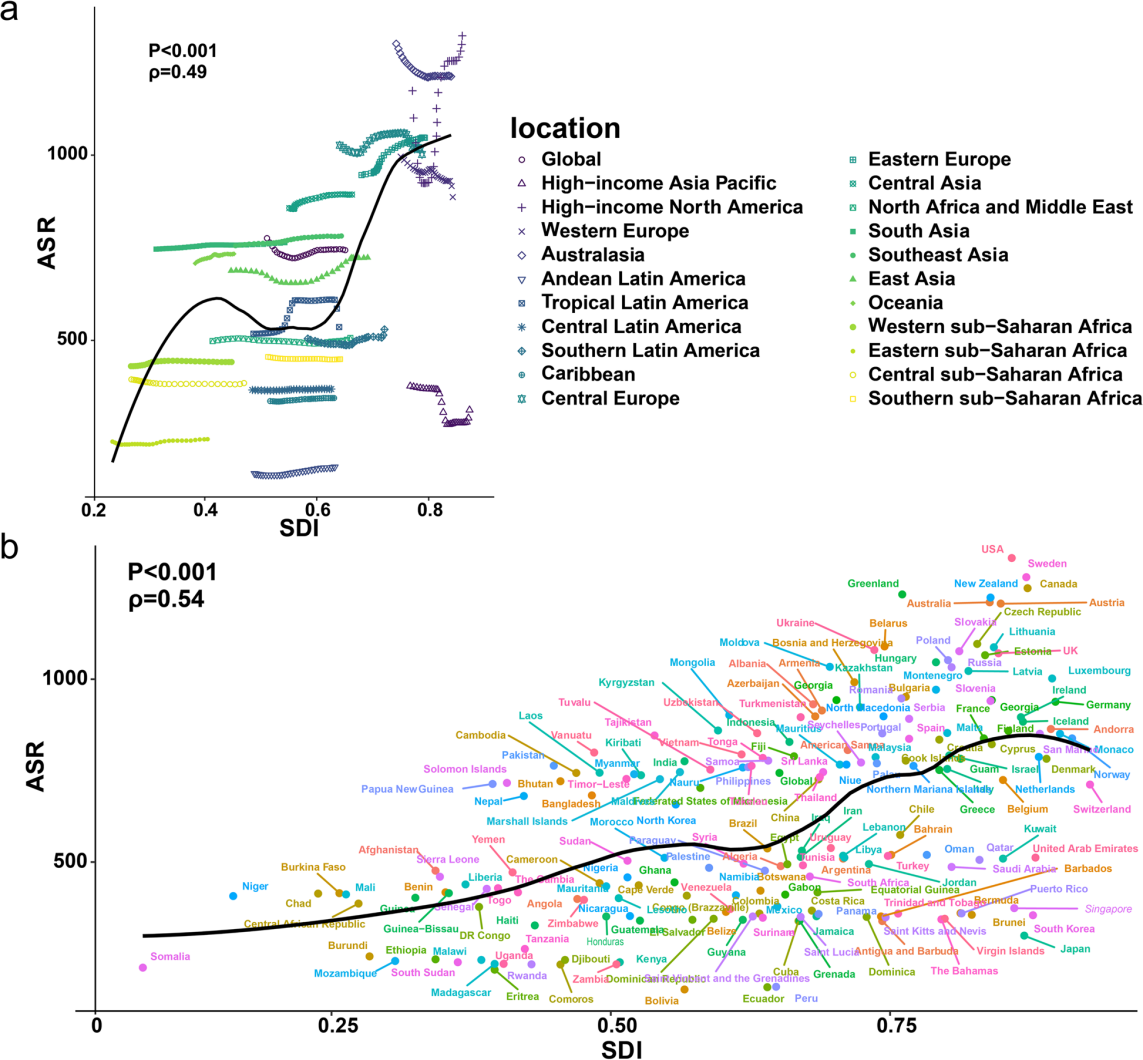
Age-standardised DALYs rates for AF/AFL for 21 GBD regions (**a**) and 204 countries and territories (**b**) by Socio-demographic Index, 1990–2019 Expected values based on Socio-demographic Index and disease rates in all locations are shown as the black line. DALYs = disability-adjusted life-years. GBD = Global Burden of Diseases, Injuries, and Risk Factors Study

The study included 204 countries and 21 regions with the 5 SDI regions to which they belonged, with significant differences in changes in disease burden across SDI groups (Fig. 5). As seen in Fig. S3, the standardized incidence, prevalence, deaths, and DALYs were higher in the middle-high and high-SDI regions, but their standardized deaths and DALYs changes showed a decreasing trend, while the standardized deaths rates and DALYs increased faster in the lower SDI regions. The prevalence of AF/AFL was still high in the high-SDI regions. Standardized prevalence rates were high in all high SDI regions except the High-income Asia Pacific. The patterns observed varied widely in many of the middle SDI regions. Some regions had little change in age-standardized rates throughout the study period, while others had fluctuating or increasing age-standardized rates, *ρ* = 0.49， *P* < 0.001 (Fig. 5a). At the global level, the age-standardized prevalence of SDI countries in 2019 showed a gradual increase with increasing SDI, *ρ* = 0.54， *P* < 0.001(Fig. 5b).

#### EAPC and ASR/HDI relationship

**Fig. 6:**
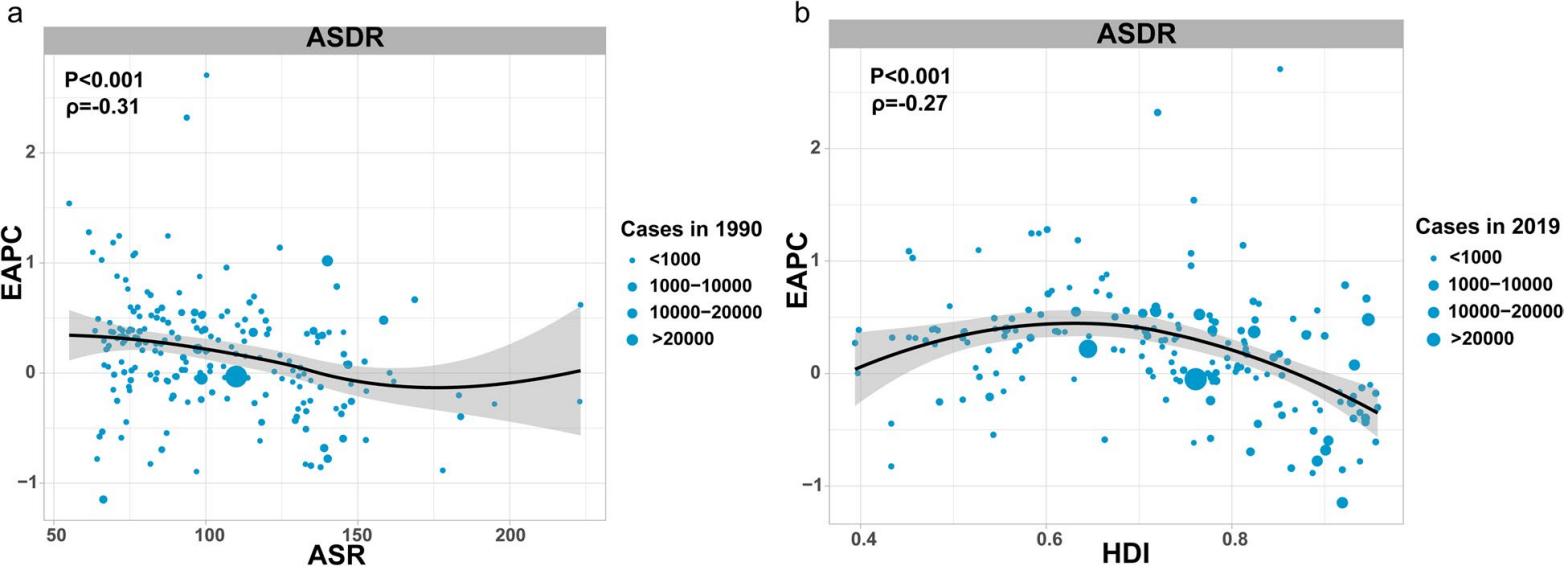
The correlation between EAPC and AF/AFL ASR in 1990 (**a**) and HDI in 2019 (**b**). The circles represent countries that were available on HDI data. The size of circle is increased with the cases of AF/AFL. The ρ indices and *p*-values presented in (**a**) and (**b**) were derived from Pearson correlation analysis. ASR, agestandardized rate; EAPC, estimated annual percentage change; HDI, human development index

As shown in Fig. 6, EAPC was significantly correlated with the Age-standardized deaths rate in 1990(ASDR 1990) and the Human Development Index in 2019(HDI 2019). ASDR 1990 for AF/AFL reflects the baseline disease reservoir, and 2019 HDI can be a proxy for the level and accessibility of health care in each country. There was a significant negative correlation between EAPC and ASDR, when ASDR was limited to less than 150/100,000 (*ρ* = − 0.26, *p* < 0.001). In contrast, this association disappeared when the ASDR was higher than 150/100,000(Fig. 6a). The EAPC of DALYs in the middle HDI region was higher than that in the low and high HDI regions, with an inverted U-shape (*ρ* = − 0.29, *p* < 0.001), countries with higher HDI experienced a decrease in ASDR for AF/AFL (Fig. 6b).

#### Decomposition analysis in DALYs

The study’s results showed that population growth between 1990 and 2019 contributed 76.60% to the increased burden of DALYs in AF, followed by aging (27.36%) and epidemiological changes (− 3.96%). The contribution of aging to total DALYs was most pronounced in the high SDI (46.72%) quintile, followed by a decreasing trend in the middle-high, middle, middle-low, and low SDI quintiles (36.36, 27.64, 21.61, and 2.13%, respectively). This is mainly driven by the growth of the population in the low SDI (89.89%) quintile. Over the analysis period 1990–2019, the epidemiological showed different trends of changes with positive contributions from the low SDI quintile, low-middle SDI quintile and middle SDI (7.98, 8.59 and 4.1%, respectively) quintile and negative contributions from the high SDI and middle-high SDI quintile (− 3.96% and − 7.89%, respectively) (Table 2).

**Table 2:** Changes in DALYs by population-level determinants from 1990 to 2019 globally and by sociodemographic index quintile

Location	Overll difference	Change due to population-level determinants (contribution to the total change)		
		Aging	Population	Epidemiological change
Global	4,605,796.75	1,259,995.307 (27.36%)	3,528,075.578 (76.6%)	− 182,274.135(−3.96%)
High SDI	1,156,001.13	540,089.803 (46.72%)	707,134.197 (61.17%)	−91,222.87(−7.89%)
High-middle SDI	1,105,410.613	401,959.109 (36.36%)	810,737.708 (73.34%)	−107,286.204(−9.71%)
Middle SDI	1,367,806.256	378,107.722 (27.64%)	933,556.264 (68.25%)	56,142.27 (4.1%)
Low-middle SDI	752,622.814	162,669.692 (21.61%)	525,298.313 (69.8%)	64,654.809 (8.59%)
Low SDI	221,757.909	4723.348 (2.13%)	199,338.15 (89.89%)	17,696.411 (7.98%)

#### Impact of AF/AFL risk factors on disease burden

**Fig. 7:**
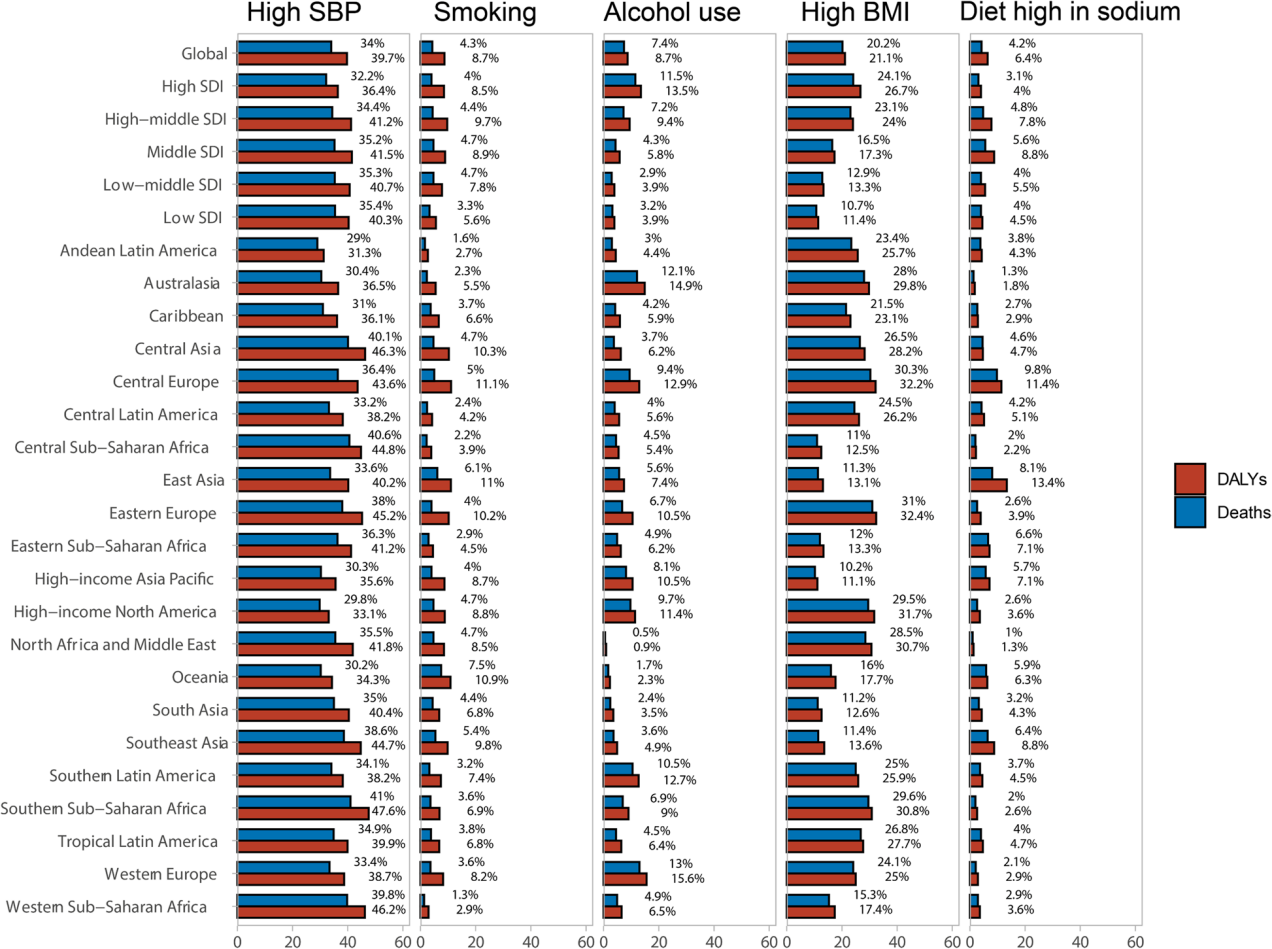
Proportion of AF/AFL DALYs and Deaths attributable to high SBP, smoking, alcohol use, high BMI, and diet high in sodium, for 21 GBD regions, SBP = systolic blood pressure. BMI = body-mass index. DALY = disability-adjusted life-year. GBD = Global Burden of Diseases, Injuries, and Risk Factors Study

At the global 2019 AF/AFL risk factor level, a substantial proportion of DALYs were attributable to the five GBD-estimated risk factors, including high systolic blood pressure 34.0% (95%UI 27.3–41.0%), high body-mass index (BMI) 20.2% (95%UI 27.3–39.9), alcohol use 19.5% (95%UI 6.3–36.0), smoking 19.1% (95%UI 4.2–34.6) and diet high in sodium 7.5% (95%UI 5.2–9.6) (Fig. 7). The effects of these risk factors varied by region. For example, the effect of high systolic blood pressure was highest in Southern Sub-Saharan Africa (47.6% of DALYs attributed to high systolic blood pressure) and Central Asia (46.2%). The effect of the high body-mass index was highest in Eastern Europe (32.3% of DALYs attributed to high BMI) and Central Europe (32.2%).

Figure S4 shows that the burden of disease due to all risk factors was highest in high SDI regions, with a standardized deaths rate of 2.5/100,000 (1.9–3.1) and standardized DALYS of 145.8/100,000 (112.9–191.1), followed by middle-high SDI regions, with a standardized deaths rate of 2.3/100,000 (1.8–2.9) and standardized DALYS of 91.8/100,000 (68.4–121.0). The middle SDI regions have standardized death rates of 2.3/100,000 (1.8–2.9) and standardized DALYs of 91.8/100,000 (68.4–121.0). Metabolic factors (high systolic blood pressure) were the most critical risk factors in all SDI regions, with a significantly higher risk share than other factors. In the low SDI, low-middle SDI, and middle SDI groups, the effect of alcohol on AF/AFL was much lower than in the high SDI group. The impact of high BMI on AF was higher in the high and middle-high SDI regions than in the low, middle-low and middle SDI regions.

As shown in Fig. S5, the risk factor level from 1990 to 2019 in the 5 SDI regions, among the 5 risk factors attributed to DALYs, no significant change was seen in the effect of alcohol. The impact of high sodium intake was gradually decreasing, with the most significant decrease in the moderate SDI regions. High BMI showed varying degrees of elevation in all SDI regions, and its baseline level was positively correlated with SDI levels. The effect of smoking showed a decreasing trend in all five SDI regions, with the most significant decrease in the high SDI region. Interestingly, in terms of high systolic blood pressure on DALYs, the trend was decreasing in the high and middle-high SDI regions, with the largest decrease in the high SDI region. The middle, middle-low and low SDI regions showed an increasing trend until 2010–2011, after which they decreased year by year but remained above their 1990 base values. The change in risk factors attributed to Deaths was nearly identical to that of Dalys.

## Discussion

This study presented a comprehensive epidemiological analysis of the global profiles of AF/AFL incidence, prevalence, deaths, and DALYs based on high-quality data. Study results show that AF/AFL burden is progressively increasing globally, with a nearly 1.1-fold increase in the number of AF/AFL prevalence and a roughly 1.7-fold increase in deaths over the past 30 years. In 2019, The number of new cases of AF/AFL was about 4.720 million, the number of prevalence was about 59.695 million. Extrapolating from the Nordpred age-period-cohort model, in the absence of effective interventions, the AF/ AFL total incidence of males will be 16.08 million, and the total number of deaths will be 1.01 million. For females, the total number of incidence will be 16.85 million, and the total number of deaths will be 1.49 million between 2030 and 2034.

Observations on global trends in the epidemiology of AF/AFL have shown that the prevalence of AF/AFL is gradually decreasing in the vast majority of European countries, which sets an example for other countries. The prevalence of AF/AFL in the Middle East and its surrounding regions is worryingly increasing at an extremely rapid rate. This may be related to the rapid local economic development in recent years, the tendency to westernize the diet, and the higher incidence of metabolic and cardiovascular diseases [17, 18]. Relevant studies have shown that patients with AF in the Middle East are younger than in Western countries and have more comorbidities. A significant number of patients with middle- and high-risk AF are not effectively treated (both for AF and for preventing its complications) [19]. The rapid economic development in the Middle East in recent years has not been matched by the slow development of education and medical care, so we can target increased spending on education and medical care and combine some of the public health approaches of European countries to improve prevention and treatment policies. Differences in the disease burden of AF/AFL also existed by gender and age, with overall incidence and prevalence higher in males than in females in 2019. Gender differences also exist at different ages, with females having a greater overall incidence than males after age 65, a greater prevalence than males after age 75, a significantly higher number of deaths than males after age 65, and a higher number of DALYs than males after age 70. It has been suggested that estrogen has a protective effect on AF by prolonging the effective atrial inactivity period, which could potentially explain the rise in the incidence of AF in females after menopause [20, 21], and it may help promote screening policies for the postmenopausal population. A European Heart Group study of 5333 people with atrial fibrillation found that females had more symptoms, such as difficulty breathing or chest pain, and lower quality of life scores. Females are significantly less likely to use rhythm control strategies (electrical cardioversion, catheter ablation, or surgical ablation) than males. This phenomenon has been observed in other studies of atrial fibrillation [22–24], showing that there may be some gender inequality in treating AF/AFL. The decline in quality of life and loss of productivity in females with the disease may increase the income gap between males and females, followed by reduced access to medical services, which helps explain the rise in Death and DALYs in females. In order to eliminate gender inequality, further research and rational allocation of resources are needed.

The burden of AF/AFL varies in different regions of economic and social development. The study showed that the burden of AF/AFL is increasing in varying degrees in all regions, but the overall deaths and DALYs ratios tend to decrease in developed regions. This may be due to the higher proportion of resources allocated to developed regions, more robust health care systems, and relatively higher levels of education among their patients, who are more active in the treatment and prevention of AF/AFL and its complications [10], why the associated deaths rates are only slightly higher despite the higher prevalence of AF/AFL in high SDI regions compared to low SDI regionr. When further analyzing the relationship between ASDR and EAPA and HDI: the EAPC of DALYs in the middle HDI regions is higher than that in the high HDI regions, with an inverted U-shape, indicating that higher HDI regions experienced a decrease in ASDR for AF/AFL and lower HDI regions experienced an increase in ASDR for AF/AFL, which corroborates the findings obtained in our SDI region. In terms of population aging, aging was the more influential factor in high and high-middle SDI regions. In contrast, population growth was mainly driven by low SDI regions (high fertility and low life expectancy). Based on the trends of Age-standardized DALYs in low and low-middle SDI regions, it is easy to see that epidemiological changes play a positive role in these regions, which is an adverse finding. In contrast, high and high-middle SDI regions have a negative role in epidemiological changes, which indicates some progress in their fight against AF/AFL. This research suggests that AF/AFL is a severe problem in less economically developed regions. It is necessary for today’s national and regional health departments to actively seek health, education, and economic-based approaches to AF/AFL management. Much of the disease burden of AF/AFL (including deaths and DALYs) can be attributed to modifiable factors such as high systolic blood pressure, high body-mass index, diet high in sodium, alcohol use, and smoking. High systolic blood pressure has the most significant impact on the disease burden of AF/AFL. There was no significant difference in the burden of AF/AFL associated with high systolic blood pressure across SDI regions in 2019. Still, the burden attributable to high systolic blood pressure was decreased in high SDI regions from 1990 to 2019, with a 20.6% reduction in standardized DALYs presentation and a 20.0% reduction in deaths. The burden is progressively increasing in regions with low and middle SDI. Studies show that exposure to higher systolic blood pressure increases the risk of AF by 19% (advantage ratio of 1.19 [1.12 to 1.27] per 10 mmHg) [25]. Several studies have shown that effective blood pressure control is beneficial in reducing the incidence of AF/AFL [26–28]. In terms of the development of the attributable impact of high BMI, its contribution to the disease burden of AF/AFL is on the rise. It has become a factor that cannot be ignored. High BMI is significantly higher in high and middle-high SDI countries than in middle-low and low-SDI countries, especially in highly developed regions such as Europe. Studies have shown that each unit increase in BMI is associated with a 4–5% increase in AF risk [29]. This phenomenon is closely related to the West’s high-calorie, high-fat, high-sugar diet. It is worth mentioning that the attributable share of smoking is showing a rapidly decreasing trend in the total AF/AFL burden, which is associated with increased global tobacco control and health promotion [30, 31]. The control of risk factors can better reduce the incidence of AF/AFL, including smoking cessation, alcohol cessation, low-salt and low-fat diet, appropriate exercise, and other healthy lifestyles. Countries at different socio-economic levels face various risk factors and should be targeted according to the characteristics of each region to develop appropriate prevention and control measures.

### Research limitations

(1). GBD2019 statistical methods may bring some shortcomings, such as the inclusion of raw data may be biased, some countries may have incomplete data information, and the quality is relatively low; (2) a country or region its internal economic development is not balanced, can not fully reflect the differences of the country or region; (3) this study mainly focuses on the epidemiological characteristics of AF/AFL analysis, lack of The analysis of some results is based on some existing related studies, and the analysis of these phenomena is not very comprehensive, such as the differences between countries with different economic development, and more targeted studies are needed to explore the weights of various factors further causing the differences; (4) In this study, the Nordpred age-period-cohort model was used to predict AF/AFL incidence and deaths without further analysis of the prevalence, which affects the accuracy of the prediction results.

## Conclusions

AF/AFL remains a major global public health problem, although the ASR of prevalence, incidence, and DALY at the worldwide level showed a decreasing trend from 1990 to 2019 (the ASR of deaths increased slightly). However, the unfavorable trend observed in this study in countries with lower SDI suggests that current prevention and treatment strategies should be reoriented. Some countries should develop more targeted and specific strategies to prevent the increase of AF/AFL.

## Supplementary Information

**Additional file 1: Fig. S1.** Projection of totaL AF/AFL incidence and death and their age-standardized rates from, 1990 TO 2034.

**Additional file 2: Fig. S2.** The number and rough ratio of incidence, prevalence, death and dalys in men and women worlwide from 1990 TO 2019.

**Additional file 3: Fig. S3.** Trends in age standardized incidence, prevalence, death and Dalys rate in 5 SDI regions, From 1990 TO 2019.

**Additional file 4: Fig. S4.** In 2019, the total rosk factors in 5 SDI regions were attributed to age-standardized death and Dalys in AF/AFL.

**Additional file 5: Fig. S5.** Trends in the 5 risk factors attributed to AF/AFL in each of the 5 SDI regions, from 1990 to 2019.

**Additional file 6: Table S1.** Nordpred model predicts the total number of Incidence and deaths of AF/AFL.

**Additional file 7: Table S2.** Nordpred model predicts the ASR of Incidence and deaths of AF/AFL.

**Additional file 8: Table S3.** Global prevalence, incidence, deaths, and DALYs of AF/AFL by age group and their rates.

**Additional file 9: Table S4.** Prevalence, incidence, deaths, and DALYs and their age-standardized rates in female AF/AFL patients worldwide, between 1990 and 2019.

**Additional file 10: Table S5.** Incidence, prevalence, deaths, and DALYs and their age-standardized rates in male AF/AFL patients worldwide, between 1990 and 2019.

## Data Availability

All data were obtained from the public open database: Global Health Data Exchange (GHDx) query tool (http://ghdx.healthdata.org/gbd-results-tool).

## Abbreviations

AF/AFL: Atrial fibrillation/flutter
GBD2019: Global Burden of Disease, Injury, and Risk Factor Study 2019
UI: Uncertainty interval
SDI: Socio-Demographic Index
ASR: Age-standardized rate
DALYs: Disability-adjusted life years
GHDx: Global Health Data Exchange
ICD: International Classification of Diseases
EAPC: Estimated annual percentage change
CI: Confidence interval
APC: Age-period-cohort
HDI: Human development index
ASDR: Age-standardized DALYs rate
