# Supplementary figures and images for: Global, regional, and national burden of disease study of atrial fibrillation/flutter, 1990–2019: results from a global burden of disease study, 2019
Source: BMC Public Health. 2022 Nov 3;22:2015. doi: 10.1186/s12889-022-14403-2 (PMC9632152; doi:10.1186/s12889-022-14403-2)

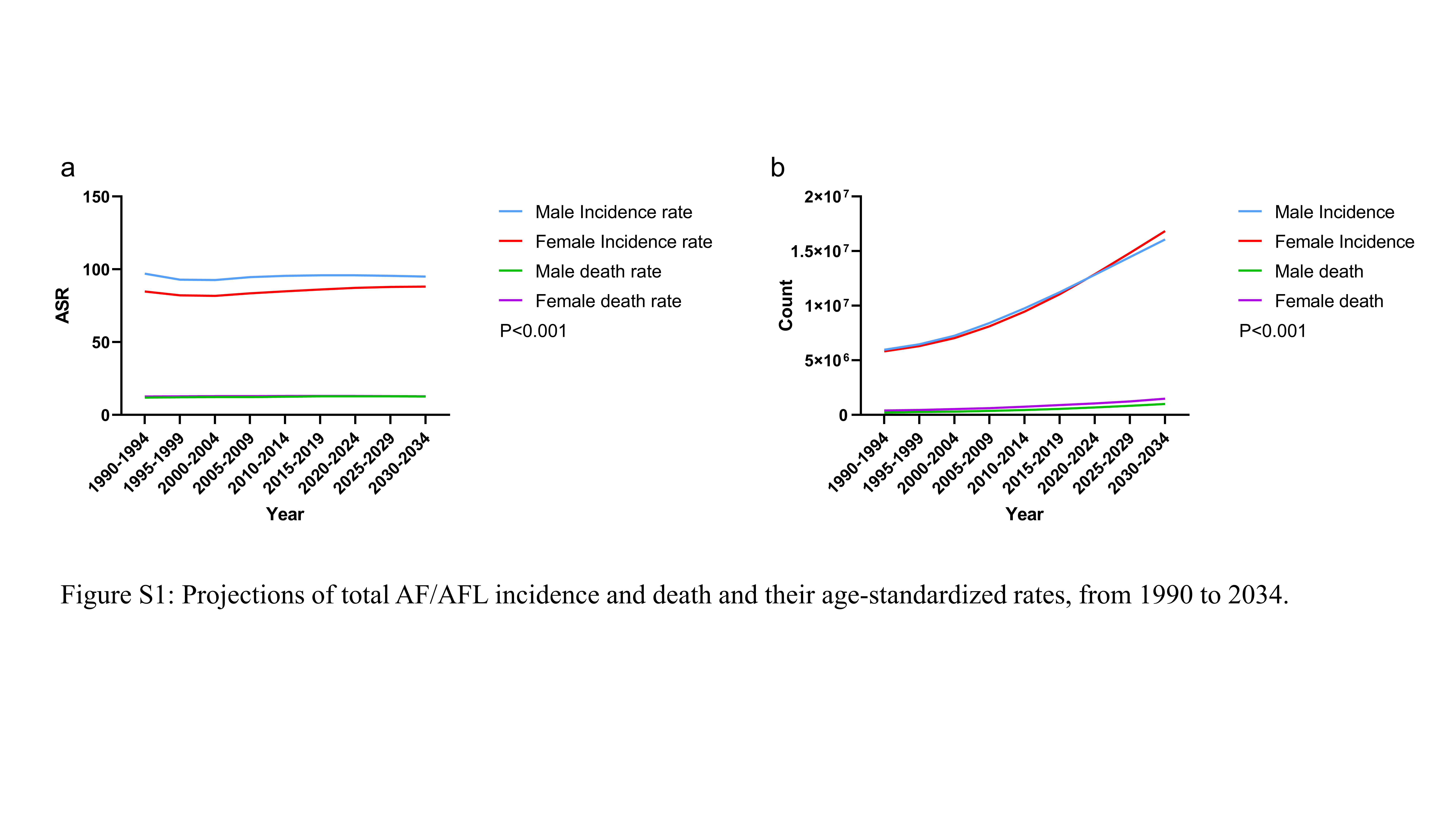

Supplement: Supplementary file 1 — Additional file 1: Fig. S1. Projection of totaL AF/AFL incidence and death and their age-standardized rates from, 1990 TO 2034. [file 12889_2022_14403_MOESM1_ESM.tif]

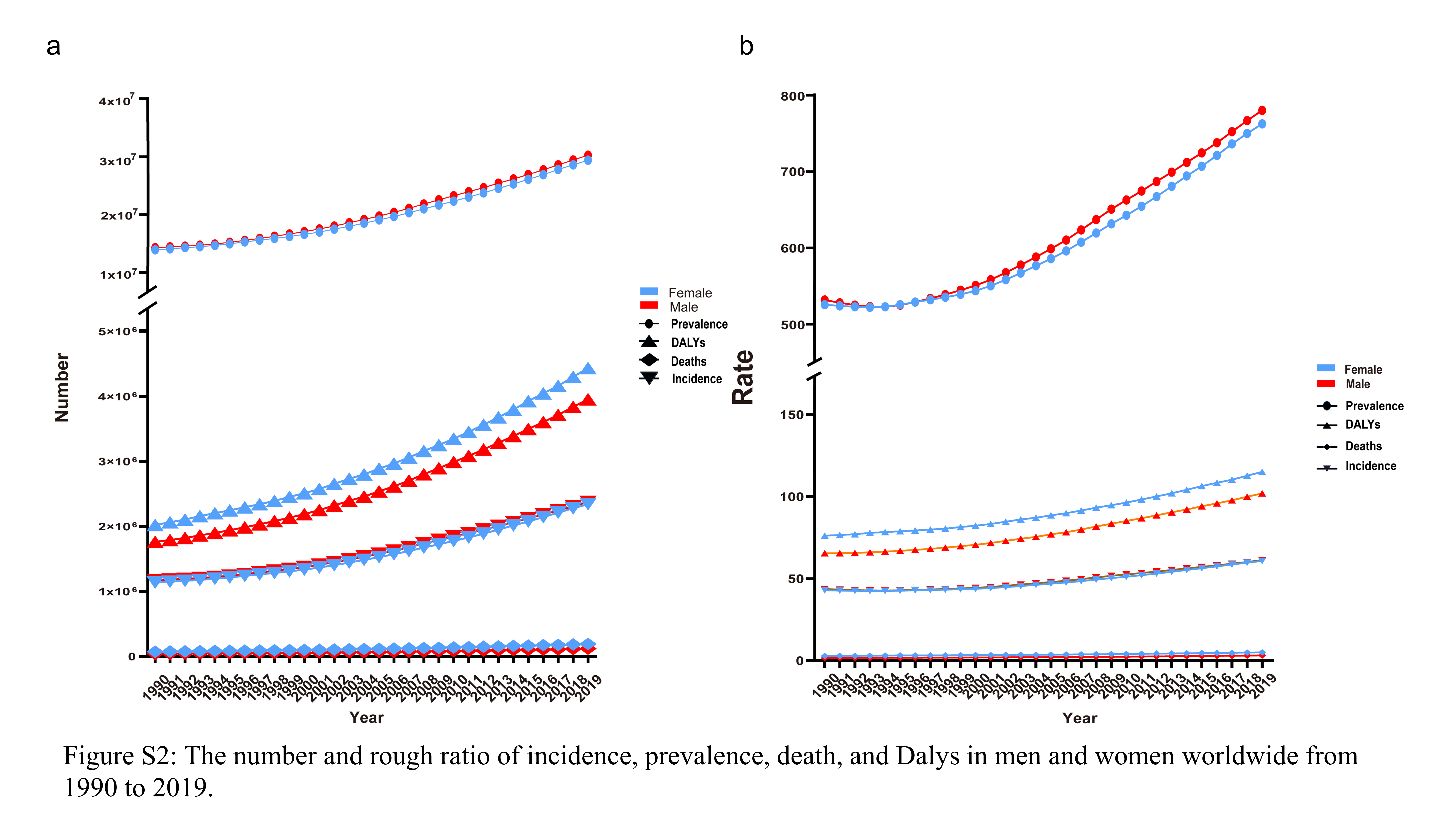

Supplement: Supplementary file 2 — Additional file 2: Fig. S2. The number and rough ratio of incidence, prevalence, death and dalys in men and women worlwide from 1990 TO 2019. [file 12889_2022_14403_MOESM2_ESM.tif]

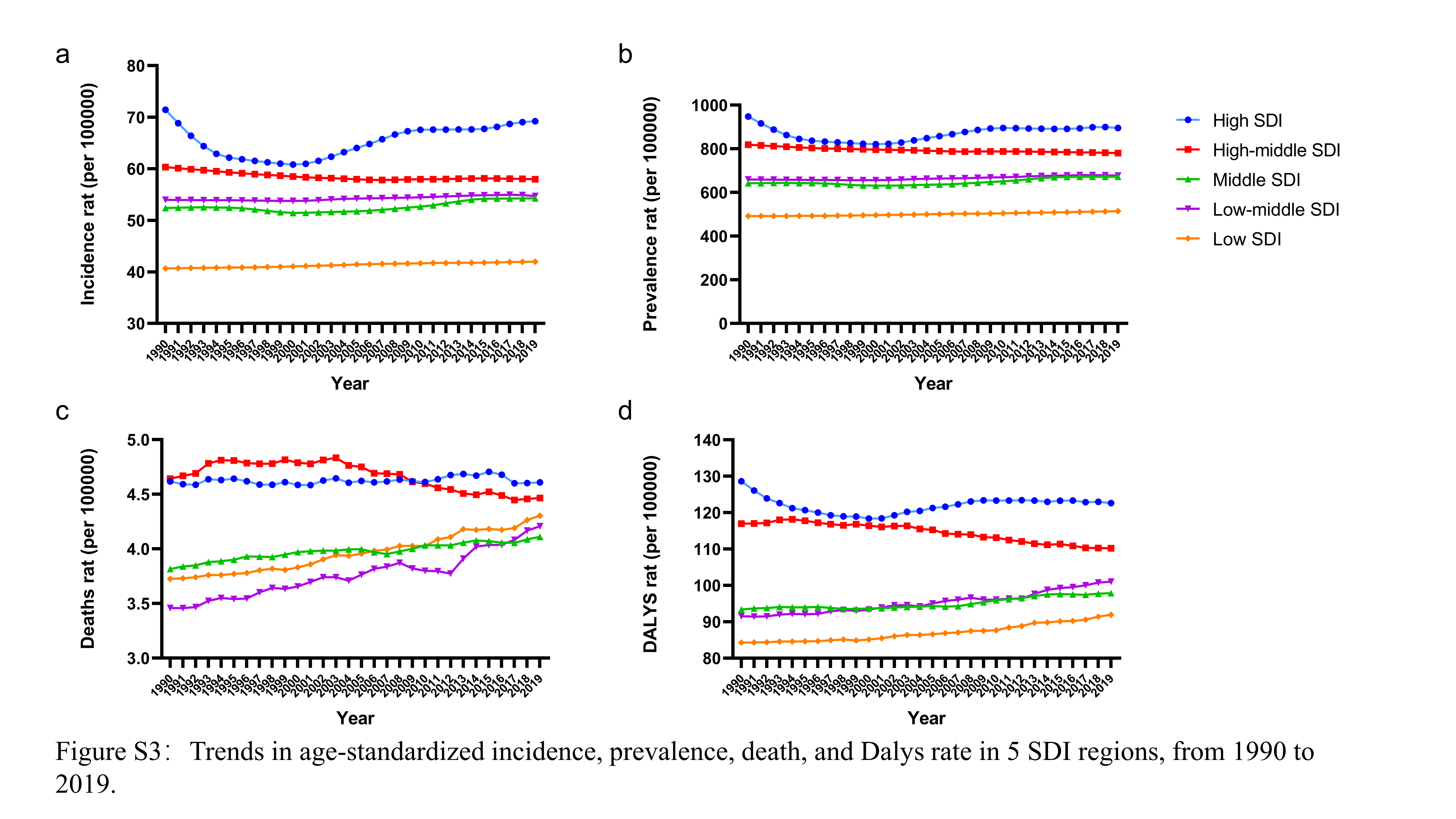

Supplement: Supplementary file 3 — Additional file 3: Fig. S3. Trends in age standardized incidence, prevalence, death and Dalys rate in 5 SDI regions, From 1990 TO 2019. [file 12889_2022_14403_MOESM3_ESM.tif]

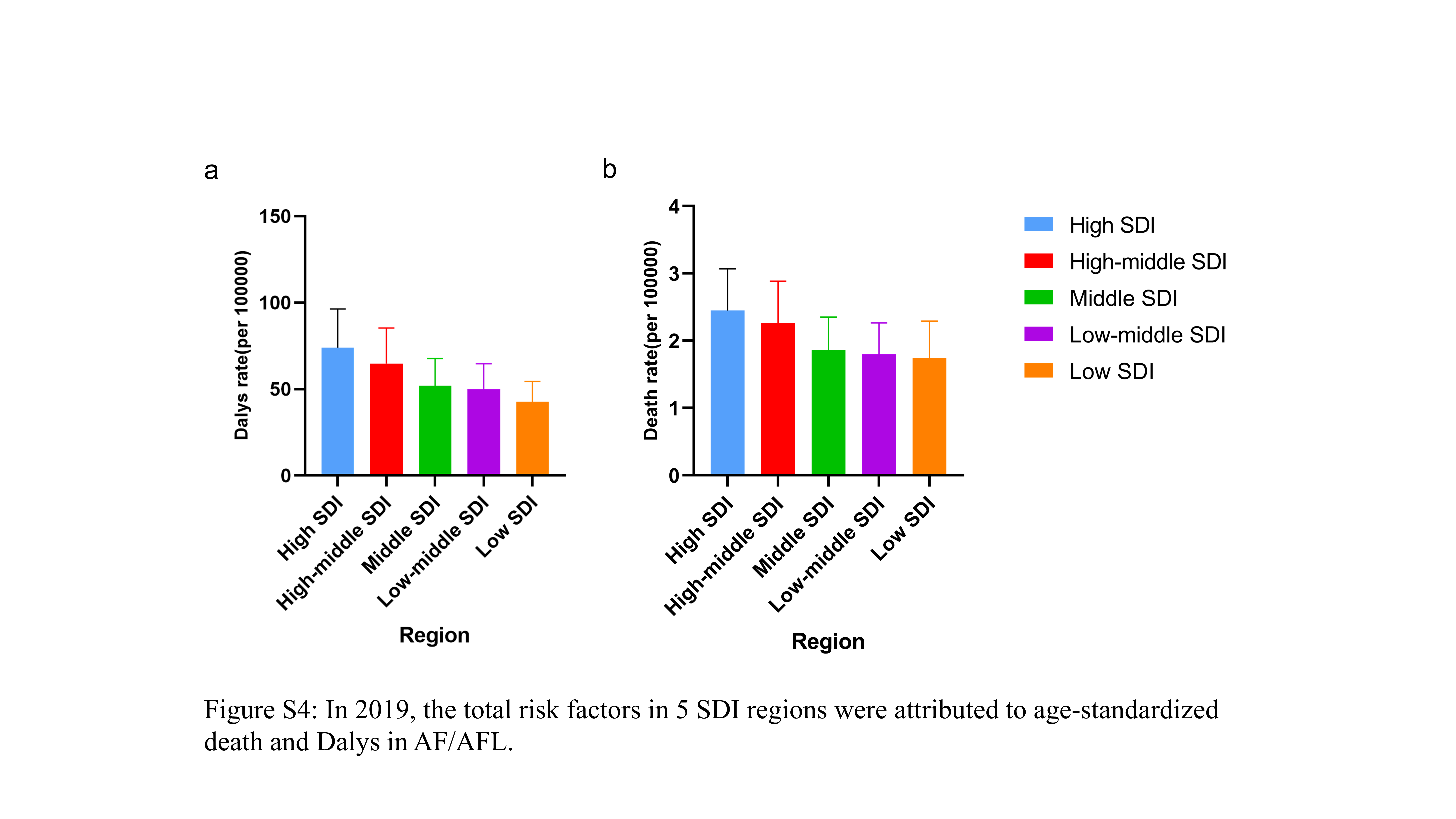

Supplement: Supplementary file 4 — Additional file 4: Fig. S4. In 2019, the total rosk factors in 5 SDI regions were attributed to age-standardized death and Dalys in AF/AFL. [file 12889_2022_14403_MOESM4_ESM.tif]

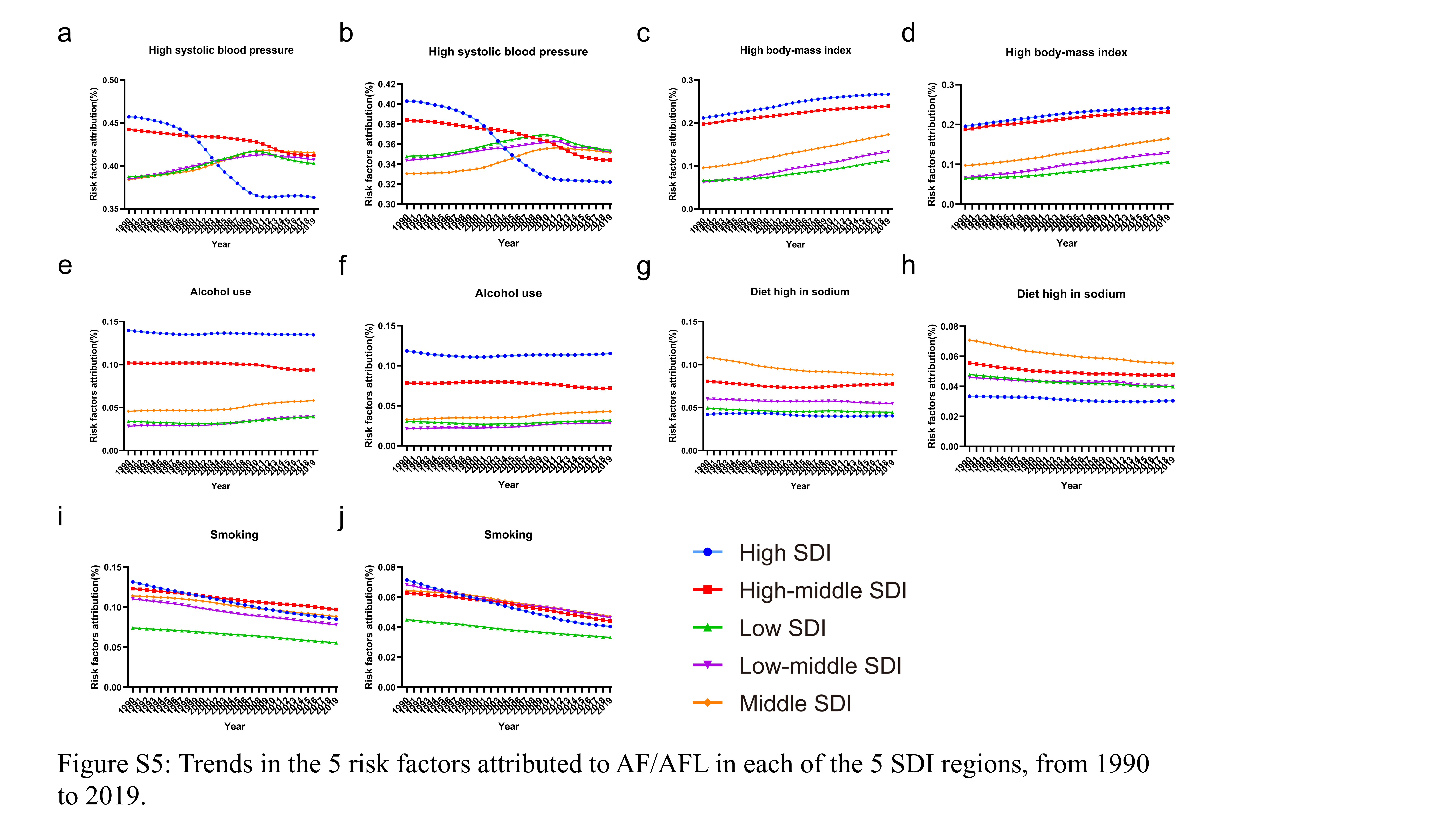

Supplement: Supplementary file 5 — Additional file 5: Fig. S5. Trends in the 5 risk factors attributed to AF/AFL in each of the 5 SDI regions, from 1990 to 2019. [file 12889_2022_14403_MOESM5_ESM.tif]
