# Supplementary material for: Global, regional, and national burden of disease study of atrial fibrillation/flutter, 1990–2019: results from a global burden of disease study, 2019
Source: BMC Public Health. 2022 Nov 3;22:2015. doi: 10.1186/s12889-022-14403-2 (PMC9632152; doi:10.1186/s12889-022-14403-2)
Supplement: Supplementary file 6 — Additional file 6: Table S1. Nordpred model predicts the total number of Incidence and deaths of AF/AFL. [file 12889_2022_14403_MOESM6_ESM.docx]

Table S1 Nordpred model predicts the total number of Incidence and deaths of AF/AFL

| Year | Incidence | | Deaths | |
| --- | --- | --- | --- | --- |
|  | Female | Male | Female | Male |
| 1990-1994 | 5826547 | 5982950 | 400264.6 | 227944.8 |
| 1995-1999 | 6314972 | 6470454 | 465541.1 | 268025.8 |
| 2000-2004 | 7040536 | 7266375 | 540288.8 | 314802.4 |
| 2005-2009 | 8124840 | 8423680 | 632289 | 375547.9 |
| 2010-2014 | 9468133 | 9772843 | 752076.4 | 460088.2 |
| 2015-2019 | 11057601 | 11244151 | 902503.6 | 562135.5 |
| 2020-2024 | 12897517 | 12838888 | 1059424 | 694987.8 |
| 2025-2029 | 14846449 | 14454435 | 1248204 | 837699.8 |
| 2030-2034 | 16846030 | 16081298 | 1490536 | 1013577 |
