# Supplementary material for: Global, regional, and national burden of disease study of atrial fibrillation/flutter, 1990–2019: results from a global burden of disease study, 2019
Source: BMC Public Health. 2022 Nov 3;22:2015. doi: 10.1186/s12889-022-14403-2 (PMC9632152; doi:10.1186/s12889-022-14403-2)
Supplement: Supplementary file 7 — Additional file 7: Table S2. Nordpred model predicts the ASR of Incidence and deaths of AF/AFL. [file 12889_2022_14403_MOESM7_ESM.docx]

Table S2 Nordpred model predicts the ASR of Incidence and deaths of AF/AFL

| Year | Incidence | | Deaths | |
| --- | --- | --- | --- | --- |
|  | Female | Male | Female | Male |
| 1990-1994 | 84.8 | 97.02 | 12.72 | 11.97 |
| 1995-1999 | 82.15 | 92.92 | 12.87 | 12.17 |
| 2000-2004 | 81.81 | 92.69 | 12.98 | 12.3 |
| 2005-2009 | 83.57 | 94.63 | 12.99 | 12.33 |
| 2010-2014 | 84.92 | 95.58 | 13.01 | 12.6 |
| 2015-2019 | 86.23 | 95.95 | 13.06 | 12.8 |
| 2020-2024 | 87.27 | 95.91 | 13.01 | 12.8 |
| 2025-2029 | 87.95 | 95.59 | 12.94 | 12.75 |
| 2030-2034 | 88.23 | 95.05 | 12.87 | 12.67 |
