# Supplementary material for: Global, regional, and national burden of disease study of atrial fibrillation/flutter, 1990–2019: results from a global burden of disease study, 2019
Source: BMC Public Health. 2022 Nov 3;22:2015. doi: 10.1186/s12889-022-14403-2 (PMC9632152; doi:10.1186/s12889-022-14403-2)
Supplement: Supplementary file 8 — Additional file 8: Table S3. Global prevalence, incidence, deaths, and DALYs of AF/AFL by age group and their rates. [file 12889_2022_14403_MOESM8_ESM.docx]

Table S3 Global prevalence, incidence, deaths, and DALYs of AF/AFL by age group and their rates

| Age | Incidence | | Prevalence | | Deaths | | DALYs | |
| --- | --- | --- | --- | --- | --- | --- | --- | --- |
|  | Number | Rate | Number | Rate | Number | Rate | Number | Rate |
| 30 to 34 | 19329.88 | 3.21 | 32858.35 | 5.46 | 93.07 | 0.02 | 8220.51 | 1.37 |
| 35 to 39 | 52549.35 | 9.71 | 204920.59 | 37.88 | 137.78 | 0.02 | 24570.13 | 4.54 |
| 40 to 44 | 117742.6 | 23.86 | 574828.0 | 116.49 | 659.27 | 0.13 | 78983.52 | 16.01 |
| 45 to 49 | 218713.8 | 46.16 | 1398125.14 | 295.09 | 1126.72 | 0.28 | 163483.4 | 34.50 |
| 50 to 54 | 375782.5 | 86.03 | 2696996.12 | 617.42 | 2167.29 | 0.50 | 302621 | 69.28 |
| 55 to 59 | 539552.4 | 145.43 | 4493711.62 | 1211.20 | 4437.48 | 1.20 | 509108.9 | 137.22 |
| 60 to 64 | 715808.6 | 229.03 | 6699534.21 | 2143.61 | 7959.62 | 2.55 | 757341.2 | 242.32 |
| 65 to 69 | 857315.8 | 331.54 | 9166489.52 | 3544.88 | 13138.85 | 5.08 | 1026504 | 396.97 |
| 70 to 74 | 742295.1 | 396.76 | 10045634.43 | 5369.50 | 23457.78 | 12.54 | 1220985 | 652.63 |
| 75 to 79 | 533591 | 419.97 | 9319687.76 | 7335.23 | 36084.42 | 28.40 | 1247481 | 981.85 |
| 80 to 84 | 334616.7 | 396.36 | 7843700.86 | 9291.03 | 65790.72 | 77.93 | 1341667 | 1589.23 |
| 85 to 89 | 147517 | 339.27 | 4708559.16 | 10829.03 | 73005.75 | 167.90 | 985999.4 | 2267.66 |
| 90 to 94 | 51006.15 | 302.57 | 1972997.77 | 11703.92 | 59524.79 | 353.10 | 547045.4 | 3245.10 |
| 95 plus | 14502.75 | 303.84 | 537039.38 | 11251.12 | 27753.22 | 581.44 | 179624.4 | 3763.18 |
