# Supplementary material for: Global, regional, and national burden of disease study of atrial fibrillation/flutter, 1990–2019: results from a global burden of disease study, 2019
Source: BMC Public Health. 2022 Nov 3;22:2015. doi: 10.1186/s12889-022-14403-2 (PMC9632152; doi:10.1186/s12889-022-14403-2)
Supplement: Supplementary file 9 — Additional file 9: Table S4. Prevalence, incidence, deaths, and DALYs and their age-standardized rates in female AF/AFL patients worldwide, between 1990 and 2019. [file 12889_2022_14403_MOESM9_ESM.docx]

Table S4 Prevalence, incidence, deaths, and DALYs and their age-standardized rates in female AF/AFL patients worldwide, between 1990 and 2019

| Year | Incidence | | Prevalence | | Deaths | | DALYs | |
| --- | --- | --- | --- | --- | --- | --- | --- | --- |
|  | Number | Reta | Number | Reta | Number | Reta | Number | Reta |
| 1990 | 1138130 | 42.85 | 13958214 | 525.52 | 74526.7 | 2.81 | 2024156 | 76.21 |
| 1991 | 1150721 | 42.68 | 14132250 | 524.18 | 77093.48 | 2.86 | 2065612 | 76.62 |
| 1992 | 1163924 | 42.55 | 14304215 | 522.91 | 79669.62 | 2.91 | 2107274 | 77.03 |
| 1993 | 1178389 | 42.47 | 14489711 | 522.25 | 83221.27 | 3.00 | 2161090 | 77.89 |
| 1994 | 1195383 | 42.48 | 14713458 | 522.93 | 85753.55 | 3.05 | 2204228 | 78.34 |
| 1995 | 1216340 | 42.63 | 14993924 | 525.51 | 88185.77 | 3.09 | 2249370 | 78.84 |
| 1996 | 1239886 | 42.85 | 15305731 | 529.02 | 90431.6 | 3.13 | 2295506 | 79.34 |
| 1997 | 1262914 | 43.05 | 15609482 | 532.12 | 92746.71 | 3.16 | 2342817 | 79.87 |
| 1998 | 1285879 | 43.24 | 15917559 | 535.32 | 95375.36 | 3.21 | 2393111 | 80.48 |
| 1999 | 1309952 | 43.47 | 16246120 | 539.11 | 98801.71 | 3.28 | 2455305 | 81.48 |
| 2000 | 1336128 | 43.75 | 16605216 | 543.76 | 101560.7 | 3.33 | 2512272 | 82.27 |
| 2001 | 1367967 | 44.21 | 17029440 | 550.34 | 104460.1 | 3.38 | 2576807 | 83.27 |
| 2002 | 1404875 | 44.81 | 17505965 | 558.34 | 108174.4 | 3.45 | 2655682 | 84.70 |
| 2003 | 1444833 | 45.48 | 18016005 | 567.14 | 111730.1 | 3.52 | 2734570 | 86.08 |
| 2004 | 1486732 | 46.20 | 18556877 | 576.62 | 114363.6 | 3.55 | 2802824 | 87.09 |
| 2005 | 1528361 | 46.88 | 19103539 | 585.99 | 118506.1 | 3.64 | 2888686 | 88.61 |
| 2006 | 1572987 | 47.63 | 19691677 | 596.24 | 122059.1 | 3.70 | 2969433 | 89.91 |
| 2007 | 1622408 | 48.48 | 20335300 | 607.68 | 126246.5 | 3.77 | 3061739 | 91.49 |
| 2008 | 1674191 | 49.39 | 21003310 | 619.58 | 130900.8 | 3.86 | 3161640 | 93.27 |
| 2009 | 1726894 | 50.31 | 21681953 | 631.63 | 134576.6 | 3.92 | 3251533 | 94.72 |
| 2010 | 1778023 | 51.16 | 22345289 | 642.92 | 139350.5 | 4.01 | 3350483 | 96.40 |
| 2011 | 1831696 | 52.06 | 23035035 | 654.64 | 144659.5 | 4.11 | 3455558 | 98.20 |
| 2012 | 1890558 | 53.07 | 23776879 | 667.49 | 150196.4 | 4.22 | 3564784 | 100.07 |
| 2013 | 1952325 | 54.15 | 24547105 | 680.84 | 155975.9 | 4.33 | 3680139 | 102.07 |
| 2014 | 2015531 | 55.25 | 25332129 | 694.39 | 161894.2 | 4.44 | 3798646 | 104.13 |
| 2015 | 2077332 | 56.29 | 26100605 | 707.21 | 168912.4 | 4.58 | 3928938 | 106.46 |
| 2016 | 2143784 | 57.42 | 26928448 | 721.29 | 174317.6 | 4.67 | 4047434 | 108.41 |
| 2017 | 2213144 | 58.62 | 27803011 | 736.38 | 179061.1 | 4.74 | 4163742 | 110.28 |
| 2018 | 2279478 | 59.73 | 28629030 | 750.14 | 186424 | 4.88 | 4302565 | 112.74 |
| 2019 | 2343863 | 60.78 | 29411703 | 762.65 | 193788.6 | 5.02 | 4437618 | 115.07 |
