# Supplementary material for: Global, regional, and national burden of disease study of atrial fibrillation/flutter, 1990–2019: results from a global burden of disease study, 2019
Source: BMC Public Health. 2022 Nov 3;22:2015. doi: 10.1186/s12889-022-14403-2 (PMC9632152; doi:10.1186/s12889-022-14403-2)
Supplement: Supplementary file 10 — Additional file 10: Table S5. Incidence, prevalence, deaths, and DALYs and their age-standardized rates in male AF/AFL patients worldwide, between 1990 and 2019. [file 12889_2022_14403_MOESM10_ESM.docx]

Table S5 Incidence, prevalence, deaths, and DALYs and their age-standardized rates in male AF/AFL patients worldwide, between 1990 and 2019

| Year | Incidence | | Prevalence | | Deaths | | DALYs | |
| --- | --- | --- | --- | --- | --- | --- | --- | --- |
|  | Number | Reta | Number | Reta | Number | Reta | Number | Reta |
| 1990 | 1175409 | 43.63 | 14327357 | 531.87 | 42511.29 | 1.58 | 1763682 | 65.47 |
| 1991 | 1184213 | 43.27 | 14453529 | 528.14 | 43902.24 | 1.60 | 1792596 | 65.50 |
| 1992 | 1194450 | 42.98 | 14594660 | 525.17 | 45369.58 | 1.63 | 1823685 | 65.62 |
| 1993 | 1206639 | 42.78 | 14757775 | 523.23 | 47293.35 | 1.68 | 1862842 | 66.05 |
| 1994 | 1222239 | 42.71 | 14964946 | 522.98 | 48868.3 | 1.71 | 1900196 | 66.41 |
| 1995 | 1242574 | 42.82 | 15239193 | 525.11 | 50532.17 | 1.74 | 1942879 | 66.95 |
| 1996 | 1267182 | 43.06 | 15570049 | 529.09 | 52016.97 | 1.77 | 1987235 | 67.53 |
| 1997 | 1293064 | 43.34 | 15923727 | 533.77 | 53481.02 | 1.79 | 2033594 | 68.17 |
| 1998 | 1319756 | 43.66 | 16291684 | 538.91 | 55120.43 | 1.82 | 2083073 | 68.91 |
| 1999 | 1347878 | 44.01 | 16679341 | 544.65 | 56875.22 | 1.86 | 2135866 | 69.74 |
| 2000 | 1376948 | 44.39 | 17082820 | 550.77 | 58640.93 | 1.89 | 2190391 | 70.62 |
| 2001 | 1410628 | 44.91 | 17538151 | 558.33 | 60733.09 | 1.93 | 2252240 | 71.70 |
| 2002 | 1449779 | 45.57 | 18053030 | 567.51 | 63155.9 | 1.99 | 2323511 | 73.04 |
| 2003 | 1492187 | 46.32 | 18602904 | 577.51 | 65297.84 | 2.03 | 2394483 | 74.33 |
| 2004 | 1536833 | 47.12 | 19185600 | 588.25 | 66974.67 | 2.05 | 2460594 | 75.44 |
| 2005 | 1581181 | 47.89 | 19772666 | 598.84 | 69727.74 | 2.11 | 2540882 | 76.95 |
| 2006 | 1628953 | 48.72 | 20409408 | 610.44 | 71956.28 | 2.15 | 2616661 | 78.26 |
| 2007 | 1682393 | 49.68 | 21119485 | 623.63 | 74742.04 | 2.21 | 2706187 | 79.91 |
| 2008 | 1737903 | 50.68 | 21853577 | 637.24 | 78091.77 | 2.28 | 2805016 | 81.79 |
| 2009 | 1793249 | 51.67 | 22587719 | 650.80 | 81030.09 | 2.33 | 2897027 | 83.47 |
| 2010 | 1845315 | 52.55 | 23282532 | 663.00 | 84537.29 | 2.41 | 2992068 | 85.20 |
| 2011 | 1897417 | 53.40 | 23972079 | 674.67 | 87965.22 | 2.48 | 3085297 | 86.83 |
| 2012 | 1952845 | 54.32 | 24699542 | 687.04 | 91746.95 | 2.55 | 3184810 | 88.59 |
| 2013 | 2009669 | 55.26 | 25439496 | 699.48 | 95944.69 | 2.64 | 3288421 | 90.42 |
| 2014 | 2067597 | 56.21 | 26193481 | 712.15 | 99894.08 | 2.72 | 3390255 | 92.17 |
| 2015 | 2124932 | 57.13 | 26946931 | 724.51 | 104140.4 | 2.80 | 3499330 | 94.09 |
| 2016 | 2184355 | 58.08 | 27748515 | 737.76 | 107908.6 | 2.87 | 3606411 | 95.89 |
| 2017 | 2247101 | 59.10 | 28611544 | 752.46 | 111759.7 | 2.94 | 3716655 | 97.74 |
| 2018 | 2311302 | 60.16 | 29460130 | 766.77 | 116778.6 | 3.04 | 3838841 | 99.92 |
| 2019 | 2376460 | 61.23 | 30283380 | 780.30 | 121548.1 | 3.13 | 3956017 | 101.93 |
